# A system for controlling vocal communication networks

**DOI:** 10.1038/s41598-021-90549-0

**Published:** 2021-05-27

**Authors:** J. Rychen, D. I. Rodrigues, T. Tomka, L. Rüttimann, H. Yamahachi, R. H. R. Hahnloser

**Affiliations:** 1 Institute of Neuroinformatics, University of Zurich and ETH Zurich, 8057 Zurich, Switzerland; 2 Neuroscience Center Zurich (ZNZ), University of Zurich and ETH Zurich, 8057 Zurich, Switzerland; 3 Porto, Portugal

**Keywords:** Developmental biology, Electrical and electronic engineering, Behavioural methods

## Abstract

Animal vocalizations serve a wide range of functions including territorial defense, courtship, social cohesion, begging, and vocal learning. Whereas many insights have been gained from observational studies and experiments using auditory stimulation, there is currently no technology available for the selective control of vocal communication in small animal groups. We developed a system for real-time control of vocal interactions among separately housed animals. The system is implemented on a field-programmable gate array (FPGA) and it allows imposing arbitrary communication networks among up to four animals. To minimize undesired transitive sound leakage, we adopted echo attenuation and sound squelching algorithms. In groups of three zebra finches, we restrict vocal communication in circular and in hierarchical networks and thereby mimic complex eavesdropping and middleman situations.

## Introduction

The exchange of information using sequences of vocally produced acoustic elements is widespread among animal species. Studies of animal communication remain challenging because the meaning of vocal signals depends not just on their sound features, but also on the behavioral state of animals and the environmental context^1–3^. As a result, the complexity of the vocal dynamics grows rapidly with group size, making it difficult to detect and assign the information conveyed to conclude causality.

A simple technique to study animal communication in a controlled setting is video and audio playback^1,4–9^. Even simple playback systems can mimic a conspecific or heterospecific individual to some degree: male zebra finches and Bengalese finches sing directed song to video presentations of female conspecifics^10^, female zebra finches perform courtship displays to videos of male conspecifics^11^, and videos of ‘audience’ hens potentiate alarm calls when produced in the presence of a predator model^5^.

Moreover, modern playback systems are capable of interacting with animals in a feedback loop^12^. These interactive playback systems (IPS), also referred to as virtual social environments (that simulate social environments) impose artificial exchanges between an animal and a robot or a computer. In songbirds, IPS have been extensively used to study social interactions and influences during developmental song learning^1,4^.

In some cases, no qualitative difference was found in the response to live versus video stimuli^3,11,13,14^. However, in other studies, an attenuated^5^ or enhanced response to video stimuli was reported^10^, suggesting that interactions among animals may exhibit dynamics that are hard to mimic using pure sound and video playback. For example, juvenile zebra finches learn better from live tutors than from interactive vocal playback^15^, indicating that some aspects of natural communication are hard to mimic using playback.

**Figure 1:**
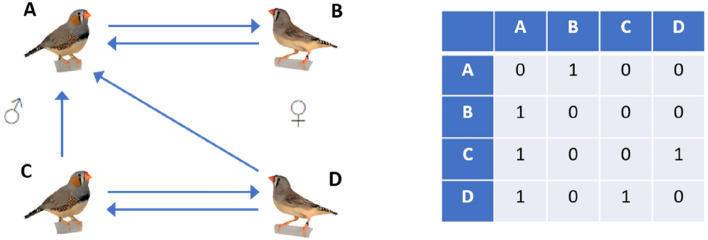
Left: schematic of a specific communication network among 4 zebra finches. In this example, the communication links within two male–female couples are symmetric, but only male A can hear the other couple. In other words, there are links from birds C and D to bird A but there is no link in the reverse direction. Right: this network can be represented as a binary 4-by-4 connection matrix in which the diagonal elements are zero and six off-diagonal elements are one.

We propose a new approach to studies of vocal communication in a naturalistic setting, which consists of connecting live animals via programmable auditory channels. The system we present allows flexible control of the communication network among up to four animals housed in separate, electronically connected sound-isolation chambers. To offer controllability of the auditory scene akin to playback systems, the auditory link between any pair of animals can be programmatically enabled or blocked in each direction independently (Fig. 1).

The main technical challenge inherent to such a communication system is to prevent transitive sound propagation in serially connected chambers. For example, in an asymmetric network C → A ↔ B in which animal B shall hear animal A but not animal C (Fig. 1), sound leakage from C to B must be prevented using a dedicated sound gating mechanism. Another challenge is to prevent acoustic feedback instabilities, which can occur in closed microphone-loudspeaker loops when the closed-loop gain is higher than 1 at any frequency.

We addressed these challenges with an echo attenuation filter and a dynamic squelch. The echo attenuation filter subtracts out a large fraction of the microphone signal elicited by the loudspeaker in the same chamber and the dynamic squelch prevents transitive sound propagation in linked chambers. Furthermore, the squelch suppresses the playback of microphone noise when the associated animal is silent. We describe the technical details of this system and present data from applications in adult male zebra finches, demonstrating reliable vocal interactions constrained by the imposed network structure.

## System description

**Figure 2:**
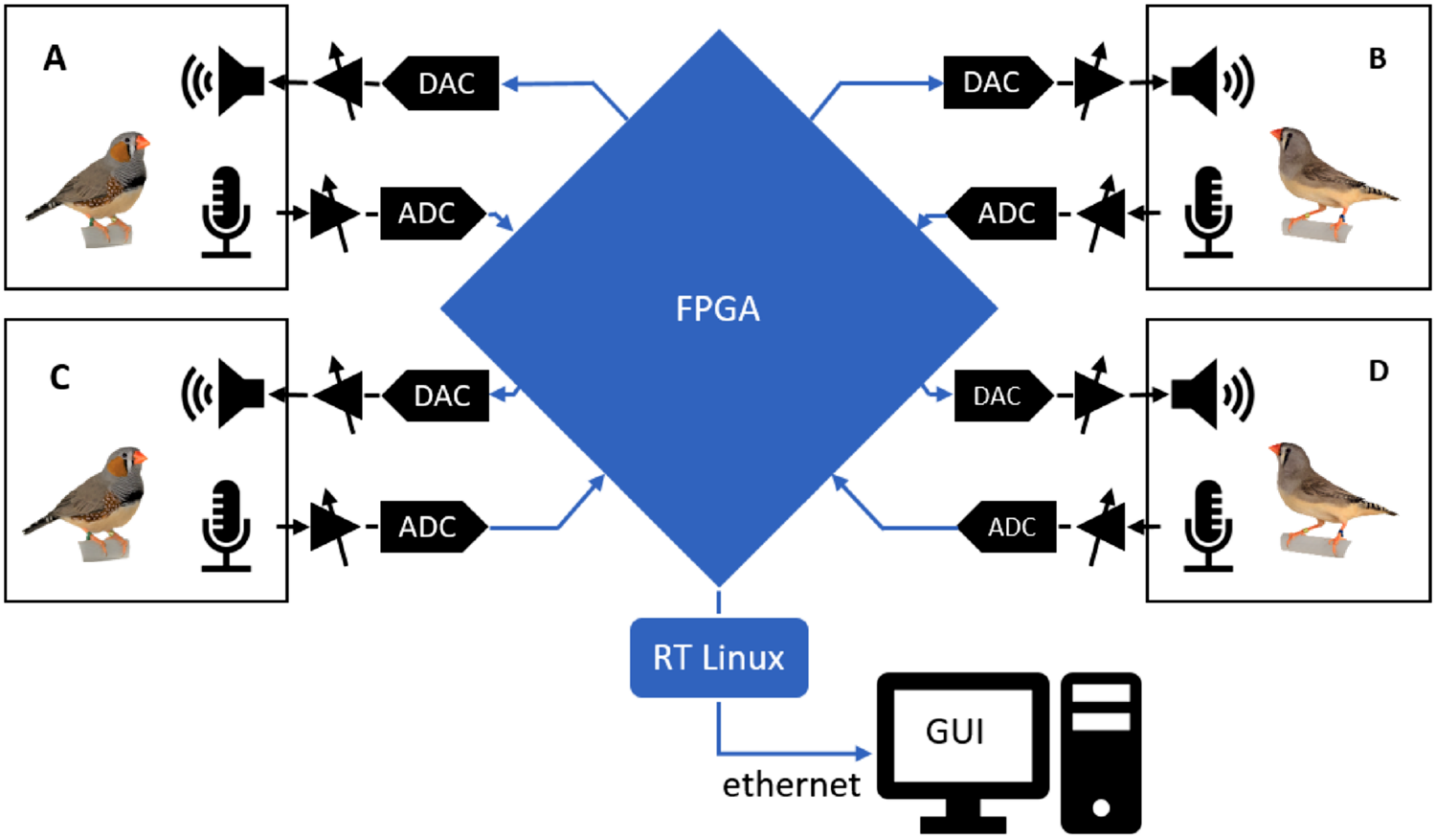
Overview of a 4-channel interactive communication system comprising analog (black) and digital (blue) components. Each bird A–D is kept in a separate sound isolation chamber equipped with a loudspeaker and a microphone. Microphone signals were amplified, digitized (ADC), and relayed to a single FPGA that processed the signals from all chambers. The digital output signals were converted back to analog signals (DAC) and were broadcast on loudspeakers in the chambers.

We implemented an interactive communication system on a compact controller (CRIO-9063, National Instruments, USA) with an FPGA (Artix-7, Xilinx, USA) programmed in LabVIEW 2018 (National Instruments, USA). The headless controller runs a real-time operating system (RT-Linux) connected over ethernet to a host computer on which system settings were defined and signals were monitored using a convenient graphical user interface (GUI, Fig. 2).

To save the data to disk, we converted all essential signals on the controller to ± 10 V analog signals (not shown in Fig. 2) and relayed them via a National Instruments data acquisition card (PCIe-6323, National Instruments) to a PC running a custom recording system written in LabVIEW.

The birds were separately housed in roughly cubic sound isolation chambers (of inside dimensions 48 cm × 48 cm × 50 cm, Industrial Acoustic Company, UK) where they were held either freely or in smaller plexiglass or wire cages (see “Technical details”).

## Chamber signal processing

**Figure 3:**
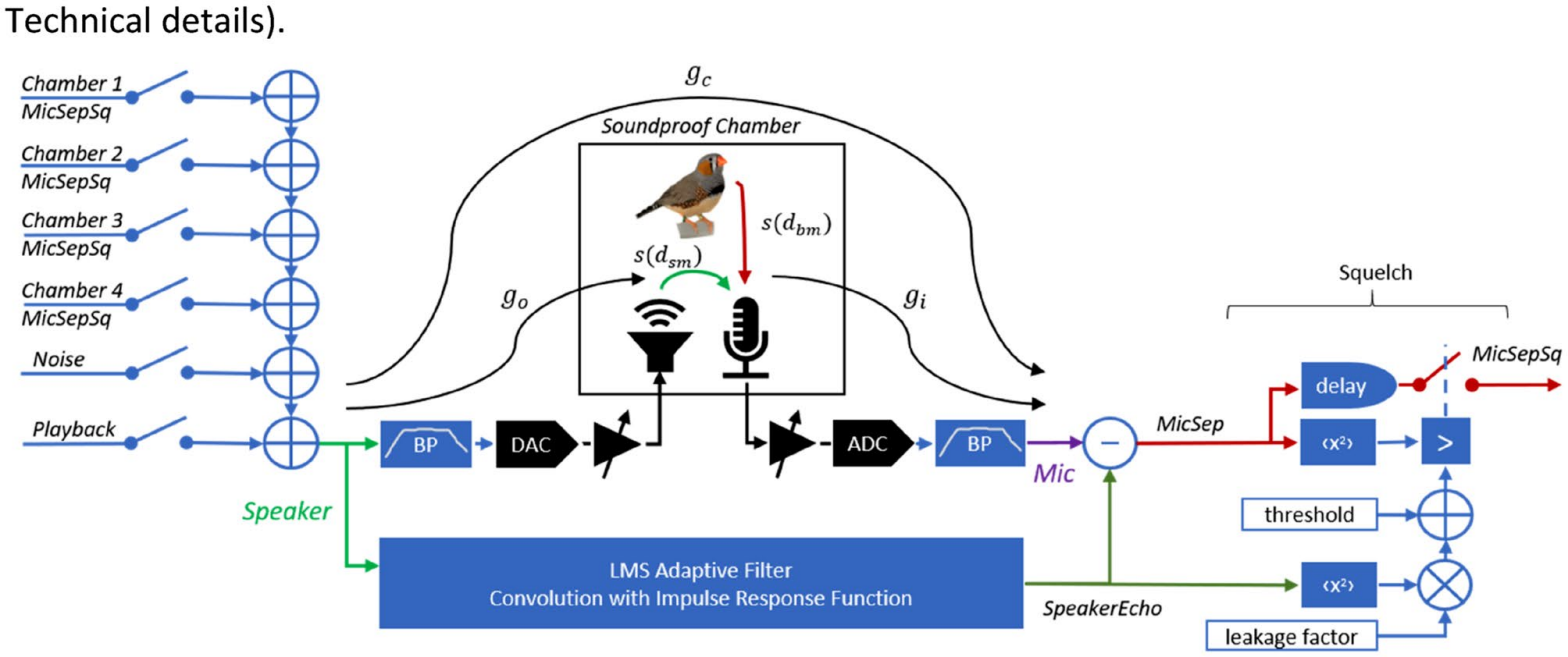
Block diagram for one chamber showing the digital signal processing blocks on the FPGA (blue) and analog devices (black). To compose the Speaker signal (green, left) destined to the loudspeaker, we selectively added signals from other chambers with playback and noise signals that we then band-pass (BP) filtered. Because the microphone picks up the signals from both the bird (red) and the loudspeaker (green), we subtracted from the Mic signal (purple) the speaker component (SpeakerEcho) that we estimated with a least mean square (LMS) adaptive filter, resulting in the separated bird signal in the chamber (MicSep). A subsequent squelch passed the MicSep signal only when its intensity was above a threshold. The threshold was the sum of a constant value and a dynamic part that was proportional to the intensity of the SpeakerEcho signal. The dynamic threshold provided robustness to unwanted sound leakage from an imperfect echo attenuation with the LMS filter. To avoid cutting vocalization onsets, we introduced a delay of the MicSep signal into the squelch. The resulting chamber output signal was the separated and squelched microphone signal (MicSepSq). Indicated by curved arrows are the gain ${\mathrm{g}}_{\mathrm{o}}$ of the output chain, the gain ${\mathrm{g}}_{\mathrm{i}}$ of the input chain, and the chamber gain ${\mathrm{g}}_{\mathrm{c}}$.

In each chamber, we mounted a microphone that we connected to a preamplifier operating at high gain. We refer to the amplified, digitized, and bandpass filtered microphone signal as the **Mic signal** (see “Technical details”). In each chamber, we placed a loudspeaker that was driven by the **Speaker signal** formed by selectively adding three types of signals (Fig. 3, “Technical details”):

1. the output signals from the connected chambers,
2. an external playback signal used to provide auditory stimuli to animals, and
3. a noise signal that was used to adapt the least mean square (LMS) echo-attenuation filter.

To prevent feedback oscillations when the communication links between chambers are engaged, we set the **chamber gain** ${g}_{c}={r}_{i}/{r}_{o}$ to values slightly less than one, where ${r}_{o}$ is the root mean square (RMS) amplitude of a white-noise Speaker signal and ${r}_{i}$ is the measured RMS Mic signal in the same chamber (Fig. 3). We tuned the value of ${g}_{c}$ to roughly − 3 dB in each chamber by manually adjusting the gain of the associated output audio amplifier. Roughly, when there is an equal distance $d$ among microphone, loudspeaker, and corresponding bird, the receiving bird hears the vocalizing bird at a loudness as if the vocalizer were at a distance of roughly $d.$

The LMS filter allowed us to estimate the **SpeakerEcho**, i.e., the part of the Mic signal that was elicited by the loudspeaker. After subtracting the SpeakerEcho from the Mic signal, we obtained the **MicSep** signal. In a final step, we blocked residual echoes on the MicSep signal using a squelch with a dynamical threshold. We termed the resulting output signal the **MicSepSq** signal (Fig. 3, “Technical details”).

## Echo attenuation with an adaptive LMS filter

To attenuate echoes, we chose a least mean square (LMS) filter that requires few computational resources and is simple to implement on an FPGA. The LMS filter is a finite impulse response (FIR) filter with adjustable coefficients. To train the filter, we generated white noise and played it on the loudspeaker, which allowed us to adjust the filter coefficients until the loudspeaker component on the MicSep signal was maximally suppressed (see “Technical details”). The resulting MicSep signal is then attributed to sounds in the chamber, i.e. the bird’s vocalizations.

To set the speed of adaptation independently of both the white noise amplitude and the filter length, the user can define a **normalized learning rate** $M$ between 0 (slow) and 1 (fast, see “Technical details”, Adaptation rate). The final FIR filter coefficients constitute our estimate of the chamber impulse response function.

Right after the adaptation process, we measure the **echo attenuation**, which is defined as the RMS ratio of Mic and MicSep signals during the white-noise presentation. We achieved typical echo attenuation values of − 30 dB. To minimize the stress in birds, we designed the white noise stimulus to be as short and soft as possible. In a series of experiments with diverse learning rates and noise levels, we identified the preferred adaptation parameters: a noise level of 65 dB sound pressure level (SPL, re 20 µP), a normalized learning rate of $M$ = 0.025, and a filter learning time of 1.5 s.

To make sure the filter coefficients converge as expected, it is important that the bird does not produce any sounds during filter training, which can be promoted by briefly turning off the lights in the chamber. Instead of verifying that the bird was indeed silent, we simply retrained the filter whenever its performance was insufficient, i.e. when it achieved an echo attenuation of less than 25 dB.

In practice, the filter performance depended on the temperature and the humidity of the air inside the chamber (because the speed of sound depends on these air properties). For example, opening and closing the chamber door and the daily temperature fluctuations can cause a degradation of up to 5 dB. To limit the degradation of echo attenuation, we automatically retrained the filter coefficients at fixed time intervals. We also found that the echo attenuation is sensitive to the placement of objects inside the chamber. The movement of the bird inside the cage can reduce the echo attenuation by 3 dB.

## Residual echo suppression with a dynamic squelch

To avoid broadcasting permanent background noise from the microphone, we implemented a gate that transmits the MicSep signal only when its intensity is above a certain threshold of typically 2 mV RMS or 38.5 dB SPL, which is 6 dB above the microphone noise. We computed the sound intensity on the FPGA using a leaky integrator with a time constant typically set to 8 ms (see “Technical details”). To not chop onsets of vocalizations, we delayed the MicSep signal by a variable time typically set to 8 ms. This delay helps to avoid broadcasting sharp and unnatural sound onsets when the MicSep signal gradually crosses the threshold during vocalization onset. The 8-ms signal delay corresponds to a sound propagation distance of 2.6 m, which is a natural auditory latency in the aviary and so should not perturb normal vocal interaction latencies (which are on the order of 100–200 ms).

In practice, the echo attenuation was not perfect, and some residual Speaker signal remained on the MicSep signal. We found that squelching the latter signal with a fixed threshold was insufficient. Namely, the softest local vocalization could be weaker on MicSep than the residual signal of the loudest remote vocalization. With a fixed threshold, we would either cut soft local vocalizations or pass leaked signals from loud remote vocalizations, either of which can be problematic.

To deal with this tradeoff and allow fine-tuning of the squelch, we designed a **dynamic squelch** that reduced the likelihood that the broadcast sounds were mis-detected as originating from the local bird. The dynamic squelch was formed by adding a variable component to the constant threshold. This variable component was given by the mean square SpeakerEcho signal (Fig. 3) multiplied by a **leakage factor**. This factor sets the tradeoff between suppressing leakage (undesired remote signal) and permitting vocal exchanges (local signal). Using such a multiplicative scheme, the squelch threshold is unaltered when no signal is broadcast, and it is raised in proportion to the estimated speaker echo signal during a broadcast. The idea of the dynamic squelch is to keep the fixed threshold low to transmit soft vocalizations from the local animal while rejecting even large-amplitude residuals from a remote animal.

## Experiments

### Symmetric and asymmetric vocal communication networks

**Figure 4:**
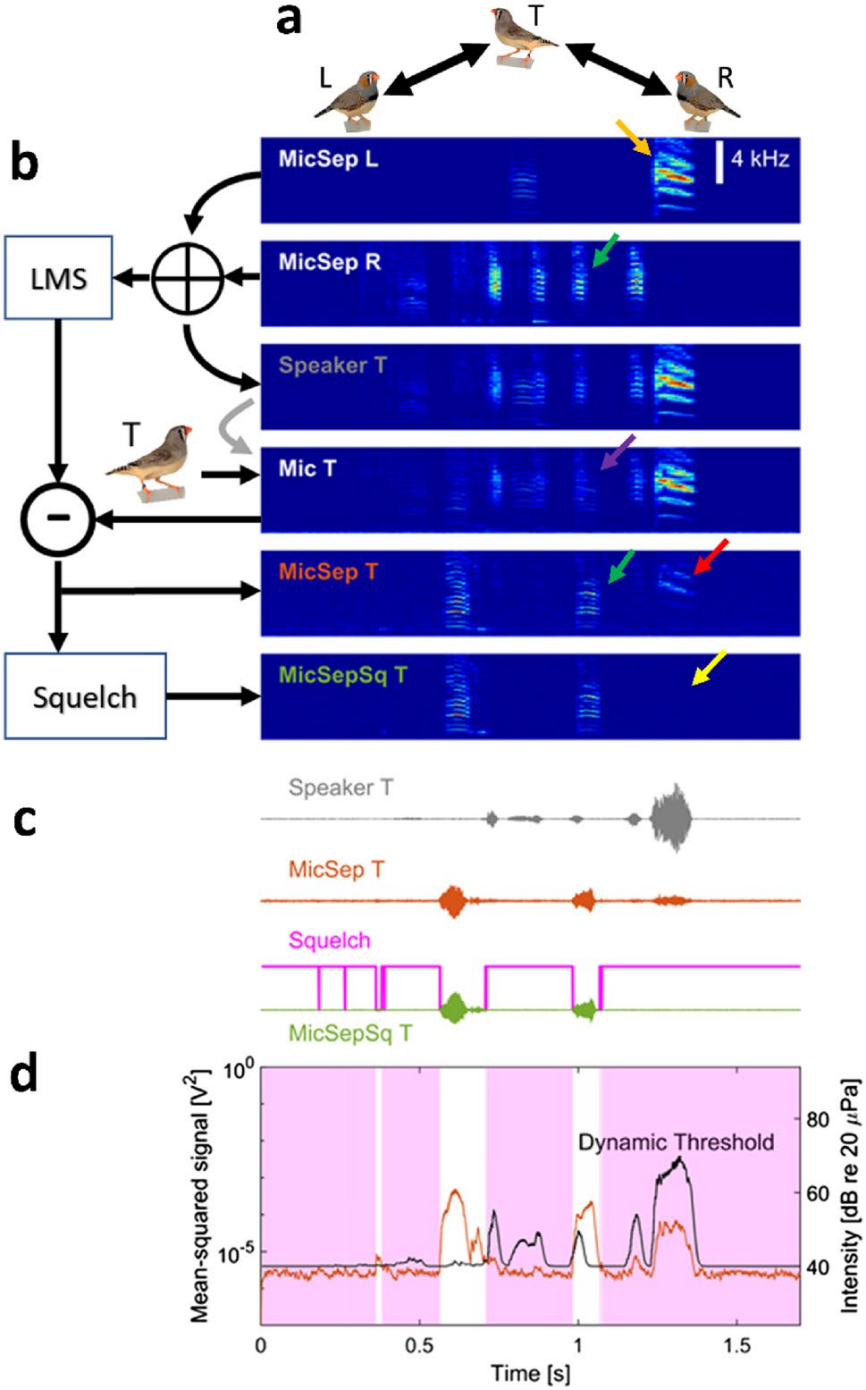
The dynamic squelch suppresses residual loudspeaker signals, demonstrated here for three birds connected in a hierarchical network. (**a**) The female T on top is bidirectionally connected (arrows) with two males L and R that are not supposed to hear each other (hierarchical network). (**b**–**d**) Example in which T and L call simultaneously (green arrows). (**b**) Even though Mic T records a superposition of both calls (magenta arrow), L’s call gets cleanly filtered out (MicSep T) by the LMS filter. In contrast, a louder call by L (orange arrow) leaves a significant echo on MicSep T (red arrow). The squelch removes the residual of that call on MicSepSq T (yellow arrow), which constitutes a clean signal, just as if L had been silent. Spectrograms have been normalized to individual color scales. (**c**,**d**) The squelch (magenta) is high when the mean-squared MicSep signal (**d**, red) falls below the dynamic threshold (**d**, black) composed of a fixed component and a dynamic component proportional to the mean-squared speaker signal. Shown are the Speaker T (gray), MicSep T (red), and MicSepSq T (green) sound waveforms. (**d**) The light magenta regions indicate times when the squelch is active and signal transmission is blocked.

For experimental validation of the echo attenuation and the squelching mechanisms, we recorded vocalizations from groups of three zebra finches connected symmetrically in a hierarchical network. We linked a female zebra finch to two separate males in a hierarchical network. The female T (top) could interact with the two males L (left) and R (right) and the males could each interact with the female but not with each other (Fig. 4a). This hierarchical network models a sort of anti-eavesdropping situation in which the female can simultaneously hear both males but not in the context of ongoing communication, which normally sets the stage for eavesdropping.

When L vocalized, the MicSepSq L signal was broadcast to T and was picked up by the microphone there (Mic T, Fig. 4b,c). This echo got attenuated by at least 25 dB (MicSep T) and the dynamic squelch ensured that the residual echo was not transmitted to R when T was quiet: no residual was visible on the MicSepSq T channel which illustrates our designed signal processing cascade.

When T vocalized simultaneously with L, Mic T picked up a superposition of both signals. The echo attenuation subtracted L’s signal to a degree that it barely left a trace on the filtered MicSep T signal, which shows that echo attenuation was effective even during simultaneous vocalizations (during which the squelch must be low to ensure T can be heard, Fig. 4).

**Figure 5:**
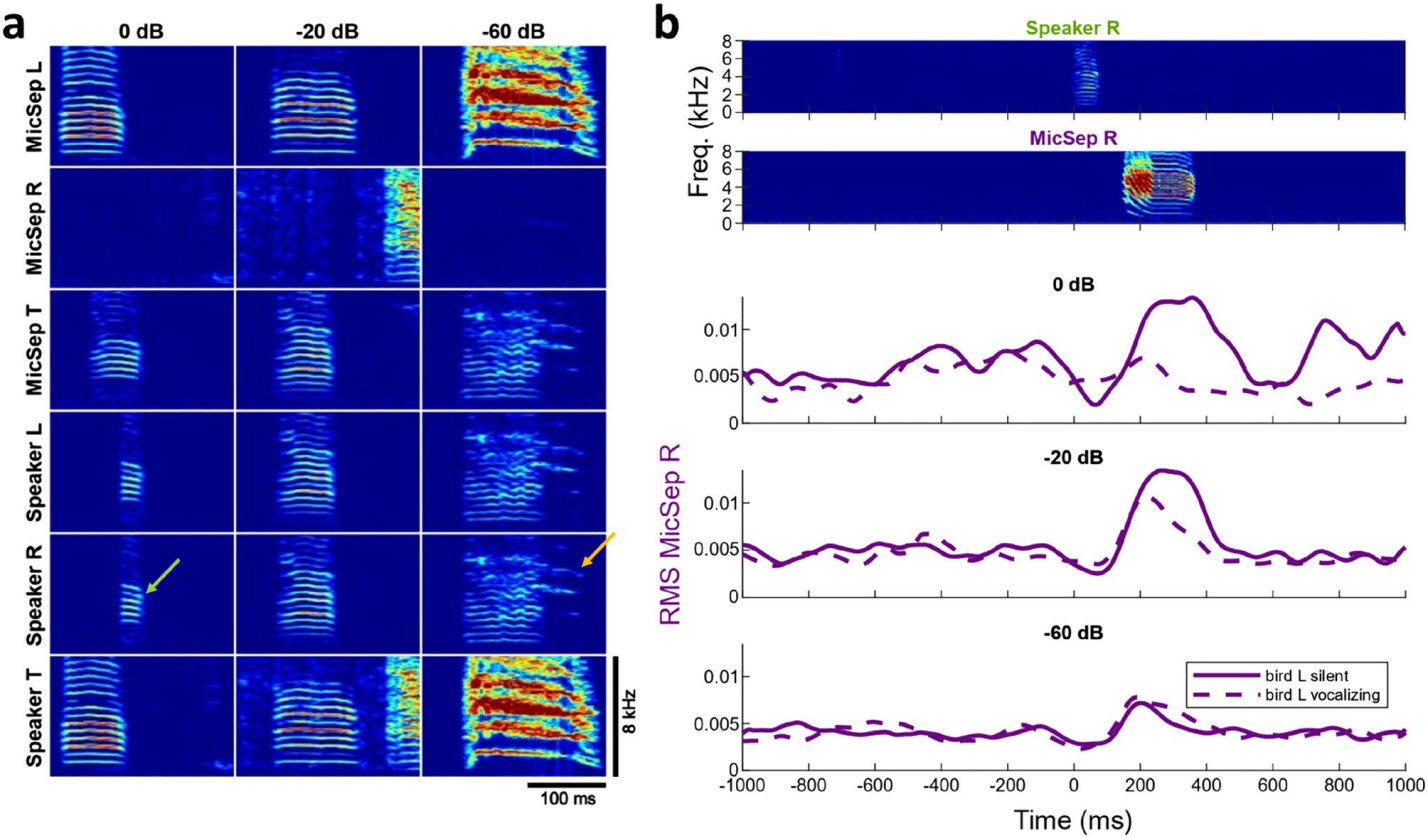
The leakage factor of the dynamic squelch sets the tradeoff between transmitting all local signals (i.e., soft calls) and suppressing leaked signals (i.e., loud residual echoes). (**a**) In a hierarchical network among 3 males, we set three different leakage factors, 0 dB (left), − 20 dB (middle), and − 60 dB (right). Shown are example spectrograms in which the top bird T produces a soft call (MicSep T) simultaneously with a loud call in L (MicSep L). A high dynamic squelch (left) produces a chopped version of T’s call on the Speaker R signal (green arrow). A midrange setting of the leakage factor (middle) preserves T’s call on Speaker R. Absence of dynamic squelching (right) produces a superposition of both calls on Speaker R, which entails that bird R can hear residual echoes of bird L (orange arrow). (**b**) Top: example spectrograms of a soft call in T (speaker R) and the response in R (MicSep R, purple) when L is silent. Bottom: root-mean square (RMS) of MicSep R signal averaged over instances in which T produces soft calls during silence (solid line) and instances in which T produces a soft call simultaneously with a loud call in L (as in a, dashed line). Time zero is the (visually detected) onset of the call in T. In case of a high leakage factor (0 dB, top), R fails to respond to concurrent calls in T (dashed line is flat). In the case of a midrange or low leakage factor setting (− 20 dB and − 60 dB), R responds similarly to both isolate and jamming calls (full and dashed lines are similar). Instances (7/540) in which T called into R’s response (in the interval [100,1000] ms) were excluded from the plot, although their inclusion had essentially no visual effect on the curves.

In this experiment, we set the leakage factor to − 20 dB, which in practice produced a good tradeoff between passing signals (vocalizations birds are allowed to hear) and removing vocalizations that should be blocked as per definition of the communication network. In a follow-up experiment, to determine the effect of sound leakage on bird behavior, we placed three males into the chambers and configured the same hierarchical network, but this time we switched among three different leakage factors, including values higher and lower than − 20 dB. When the leakage factor was very high (0 dB), we observed that soft calls by the bird at the top of the hierarchy (T) got chopped by the squelch when they co-occurred with broadcasts of loud calls in L (Speaker R and L, Fig. 5a, left). The consequence was that R diminished its responses to these chopped calls compared to when T produced the soft calls while L was silent (Fig. 5b, top curves). Thus, a high setting of the leakage factor can negatively impact superposed calls and lead to reduced responses in the receiver.

When the leakage factor was set to an intermediate value (− 20 dB, Fig. 5a, middle), the signal chopping occurred only very rarely and R’s responses to T’s calls barely depended on whether the latter were produced during silence or simultaneously with a call in L (Fig. 5b, middle curves).

Finally, when the leakage factor was set to a low value (− 60 dB), no chopping occurred and the responses in R did not depend on whether T’s calls were isolated or superposed (Fig. 5b, bottom curves). However, under this low setting, R could un-intentionally eavesdrop on calls from L, especially when the latter were loud and produced a large residual echo (Fig. 5a, right). To avoid this latter situation, for the remaining experiments, we set the leakage factor to − 20 dB.

### Communication networks constrain vocal interactions

In subsequent experiments, we tested whether birds engaged in reliable vocal interactions constrained by the network topology.

We imposed on the vocal interactions two distinct networks, either a symmetric hierarchical network as before, or an asymmetric ring network that we judged was sufficiently different from the hierarchical network to observe an effect of topology. The ring network models a middleman communication situation, in which message passing between any pair of birds is direct in one direction but indirect in the other.

**Figure 6:**
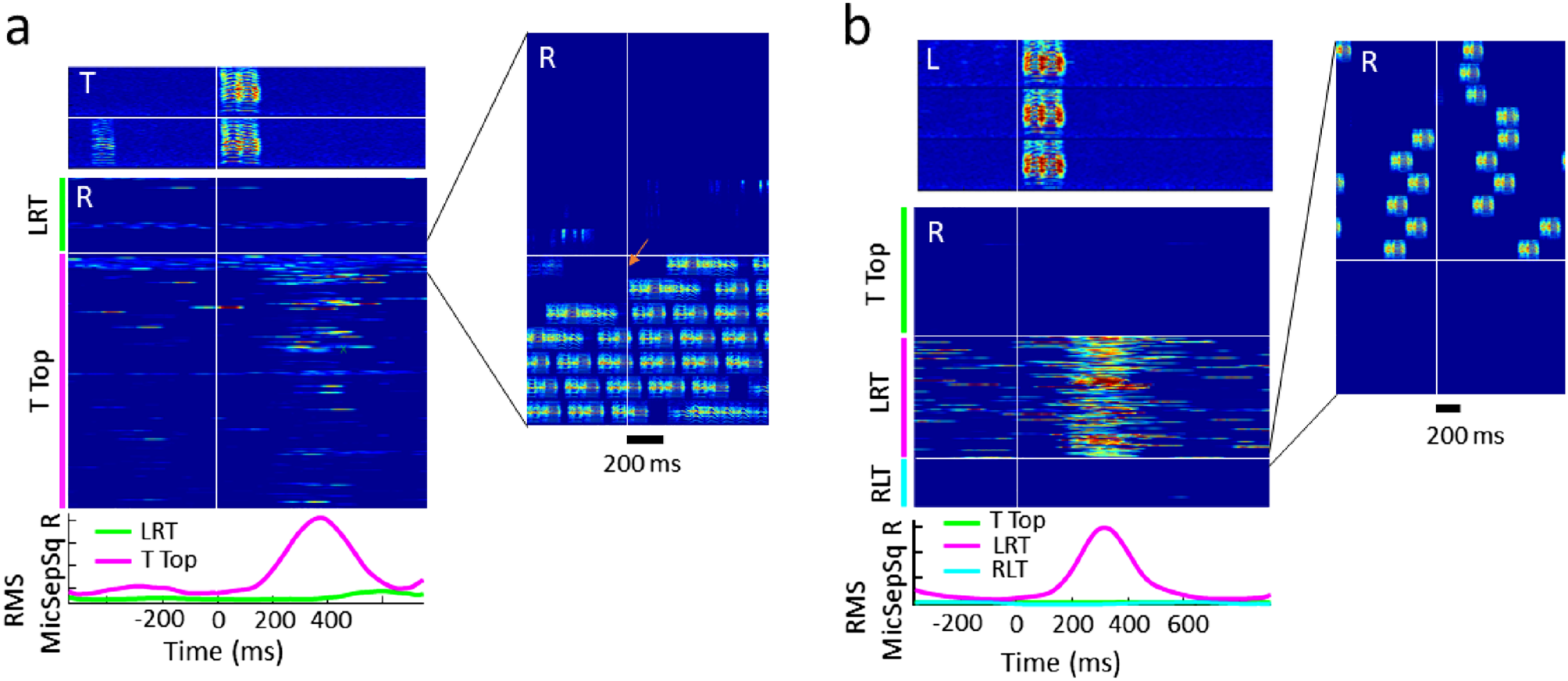
The communication network constrains vocal interactions. Vocal interactions can change very sensitively in response to switches between communication networks, shown here for hierarchical and cyclic networks. (**a**) Switch from L=>R=>T=>L (cyclic, LRT) to L<==>T<==>R (hierarchical, T top) leads to an initial string of call response in R to the first audible call in T (orange arrow in inset). Shown are spectrograms of two example calls in T (top) and several hundred responses in R depicted as a root-mean-square (RMS) stack plot (middle), aligned to the onset of the calls in T when T’s calls are not audible (LRT, top) and when they are (T top, bottom). The calling times in T run from top to bottom. The bottom curves represent the RMS MicSepSq R curves averaged over all calls in T in both conditions. R responds with a latency in the range 300–500 ms, with waning reliability. The inset on the right depicts spectrograms of responses in R right before the network switch (top) and right after the switch (bottom), aligned to the onsets of T’s calls (vertical white line). Both T and R produce dense strings of calls, which leads to multiple depictions of a given call in R in subsequent rows. (**b**) Switch from L<==>T<==>R (hierarchical, T top) to L=>R=>T=>L (cyclic, LRT) uncovers vigorous responses in R to calls in L (example spectrograms on top). However, L does not respond to R when the cyclic network changes direction to R=>L=>T=>R (cyclic, RLT). The inset on the right illustrates the immediate silence following the switch to the second cyclic network.

In one experiment among 3 males, we found that switches between the hierarchical and the cyclic networks triggered strong changes in vocal interactions (Fig. 6). Adding a feedback connection to a unidirectionally connected bird pair, from R=> T (cyclic) to R<==>T (hierarchical) could result in rapid and vigorous vocal responses in R right after the first call in T that was audible to R (Fig. 6a). Conversely, when switching from hierarchical to cyclic, at the most extreme case of two birds at the bottom of the hierarchy (L and R), one bird switched from not responding to a single call of a given type when not connected (hierarchical) to responding to virtually every single call of that type when the cyclic connection appeared (Fig. 6b), demonstrating that animals can react dramatically to imposed network changes.

**Figure 7:**
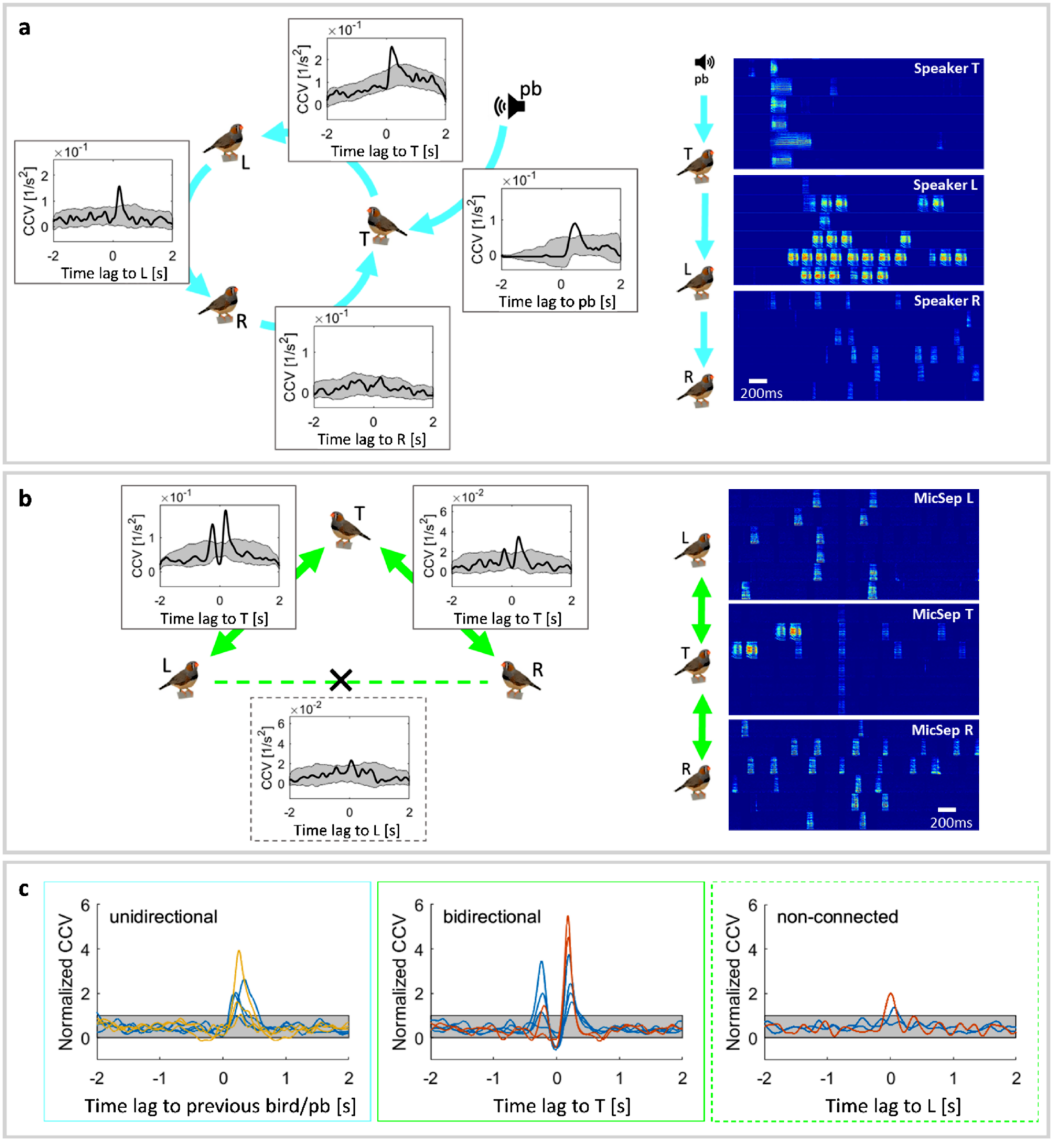
The structure of vocal interactions mirrors that of the imposed network. (**a**) In an asymmetric network, the interactions tend to be asymmetric, and (**b**) in a symmetric network, they tend to be symmetric. (**a**) In pairs of asymmetrically connected birds, cross-covariance (CCV) functions (black lines, see “Technical details”) indicate unidirectional vocal exchanges revealed by unimodal peaks. Stacks of example spectrograms are shown (right) with the auditory stimulus (pb) presented in chamber T (top), T’s response broadcast to bird L (middle), and L’s response broadcast to R (bottom); corresponding rows in the 3 sub-panels are from simultaneous recordings. (**b**) In a symmetric hierarchical network, CCV functions reveal bidirectional vocal interactions. (**a**,**b**) The gray areas represent 3 standard deviations of a random shuffle predictor (see “Technical details”). (**c**) Normalized CCV functions in unidirectional (left), bidirectional (middle), and non-connected (right) bird pairs, n = 3 bird groups (blue in all 3 subpanels, orange in the middle and right subpanel, and yellow in the left subpanel) across a total of 5 different network configurations (two asymmetric, three symmetric). To allow for comparison among experiments, CCV functions were normalized by the shuffle predictor (gray areas; see “Technical details”), which sets the threshold for statistical significance at a fixed unit distance along the y axis.

We quantified the reliability of call–call interactions in pairs of birds in terms of the cross-covariance (CCV) function (see “Technical details”). We found that a connection from bird A to bird B typically entailed the presence of reliable vocal responses in B to calls in A: the CCV function often peaked above a shuffle predictor (corresponding to p < 0.01, see “Technical details”). As expected, when connections were unidirectional, the CCV functions displayed at most a single peak at a positive time lag (Fig. 7a,c), in agreement with the causality imposed by the network.

Pairs of disconnected birds can be prevented from hearing each other and from direct vocal interactions by appropriate separation and sound isolation of recording chambers. Nevertheless, calls in non-connected birds could be correlated as shown in Fig. 7b,c, in which two birds L and R at the bottom of a hierarchical network exhibited a CCV peak near a zero-time lag, indicating that both birds tended to respond to the same calls in T. Such observation illustrates the well-known fact that correlation does not imply causation, because correlations can arise from a common cause, i.e. bird T at the top of the hierarchy. We observed such non-causal correlations in 2/3 non-connected bird pairs at the bottom of the hierarchy.

The same was not observed in bidirectionally coupled bird pairs (L<==>T and R<==>T). In 4/6 of such pairs, we observed two significant CCV peaks: one at a negative time lag (bird T responds) and one at a positive time lag (bird T is responded to). Such symmetric interactions are characteristic of turn-taking, which is typical in many species including zebra finches^13,16–18^. Moreover, in 5/6 bidirectionally connected bird pairs, the birds on top of the hierarchy were less responsive (average normalized CCV peak 1.56) than the lower birds (average peak 3.31), suggesting that a larger social network entails less reliable communication.

## Discussion

Using standard off-the-shelf components, we implemented a digital system for controlling the vocal communication network among a small group of animals. The system yields high-quality recordings of each animal’s vocalizations, provided the animals are separately housed in acoustically distinct environments. In experiments using zebra finches, we demonstrated two key features, which are (1) echo attenuation to prevent feedback instabilities and (2) a dynamic squelch that in restricted communication networks provides control over the amount of transitive sound propagation. We discuss both these features in the following.

Echo attenuators are part of virtually all speaker-based telecommunication systems, their need arises when two or more vocalizers are connected in a closed-loop. To train the echo attenuation filters with minimal impact on birds, we identified the least burdensome stimulus parameters (in terms of intensity and duration). In standard experimental cages for small songbirds, we achieved a satisfactory 30-dB echo suppression.

Echo attenuation has in the past enabled studies of the effect of communication on the behavior of individuals within their isolated environments. A two-channel version of the system presented here has enabled targeted control of auditory input in a study of learning from observation^19^. In that study, a demonstrator bird was separated from an observer bird; while the demonstrator was engaged in learning an auditory discrimination task from aversive reinforcement, the observer could gain information about the auditory stimuli and the demonstrator’s behavior. By briefly blocking the communication link during the aversive reinforcement, it was possible to pin down the role of hearing on the learning outcome in the observer, which would not have been possible if animals had been housed together. Thus, already simple two-animal communication systems based merely on echo attenuation are suitable for addressing relevant questions about social learning.

More elaborate questions can be answered in communication networks comprising three or more individuals. We have shown that in networks with partial connectivity among three or more animals, an additional challenge arises, which is leakage or transitive sound transmission. To address this issue, we proposed squelching as a viable approach. We showed that a dynamic squelch suppresses sound leakage, though it introduces the caveat of chopping some soft vocalizations when these coincide with a loud broadcast. Our observations that chopped vocalizations (0 dB dynamic squelching) suppress responses whereas un-chopped and overlapping vocalizations (− 20 dB and − 60 dB dynamic squelching) do not, will need further testing in future experiments, in which chopping is observed.

We provide no universally applicable recommendation on how to deal with the tradeoff between sound leakage and sound chopping during vocal collisions. In general, we would advise setting the dynamic squelch such as to minimally impact the scientific question studied. On the one hand, in controlled tutoring experiments, in which transitive propagation is detrimental because juveniles need to be acoustically isolated from a tutor, a high setting of the leakage factor is advisable. On the other hand, in experiments in which all types of vocal exchanges among a subset of birds are to be studied, sound chopping should be made a rare event. Thus, the detailed use of our system will dictate the appropriate level of dynamic squelching.

We restricted vocal exchanges to diverse sub-networks and thereby regulated the social complexity among animals. The communication networks we imposed were sufficient to enable non-trivial vocal exchanges that were not merely reflexive but reflected birds’ personalities or states (Fig. 6), and ranks in the group (Fig. 7). As such, there are many possible uses for our system when applied to three or more birds. For example, our system could complement observational approaches using small backpack recorders attached to animals^20–22^. That is, our system can help to overcome a shortcoming of observational studies, which can merely yield hypotheses about the ‘meanings’ of certain types of vocal interactions but are not amenable to selective testing of these hypotheses because vocal exchanges among animals are virtually impossible to manipulate without a dedicated communication system. Thus, when a certain meaning has been hypothesized from observation in freely interacting animals, it would be reassuring to infer the same meaning in loss-of-function (removed connection) and gain-of-function (e.g. playback) experiments implemented with our system.

There are several limitations of our system, which could be addressed in future extensions. For example, it is currently not possible to manipulate sound direction because we use only one loudspeaker per chamber. Birds can estimate sound source direction from interaural time differences (ITDs) and interaural level differences (ILDs). We could manipulate these cues to some degree by using a distinct speaker for each link in the network, in which case, in a network of 4 birds, we would need up to 12 speakers, 3 in each chamber. Accordingly, we would need to calculate up to 12 LMS filters in total, which would mildly increase the complexity of our hardware and software architecture.

Although we digitized only the acoustic communication mode, it is a simple matter to digitize the visual communication channel using cameras and computer screens. Advances in generative modeling of animal imagery^23,24^ could open the door to countless possibilities such as artificial visual societies. In combination, combined audio-visual communication systems could provide a means to play evolutionary games.

Because we make use of a powerful FPGA, additional signal processing is possible to enhance the function of the system. For example, we could add routines for real-time detection of a certain syllable^25^ and computation of its pitch. Such processing is required in operant conditioning experiments in which birds adapt the pitch of their syllables^26^. In our context, selective pitch estimation would allow us to study the role of pitch and its adaptation in a social context. Even a vocoder could be implemented that shifts the pitch in real-time^27^, which would allow studying the effect of pitch variability on the receiver bird.

The system as described is laid out for the hearing range of zebra finches. By using different microphones and loudspeakers, the signal range could be expanded. As a result, many species could be studied that vocalize in the ultrasonic range, such as bats^28^, rodents^29^, and frogs^30^. In terms of signal processing, the ultrasonic range is more challenging to work with because the sampling rate must be higher. Also conceivable are extensions to underwater environments. For example, interactions among cetaceans could be experimentally examined by keeping animals in separate pools. Such a setup has been proposed as enrichment for captive cetaceans^31^. The squelch could play an important role in such an application because playback experiments have shown that cetaceans react to even soft noises^32^. In the free-range and under-water setting, echo cancelation filters may need to be much longer (because sounds propagate much further in water), which should be well possible with our chosen system architecture.

Last but not least, instead of merely switching a binary connection matrix, the connection links could be more finely manipulated using a gain and a delay, with the result of simulating virtual distances between animals. Because acoustic communication evolved to be useful over large distances and without visual contact, experimental manipulation of virtual distance can be useful^33,34^. Furthermore, adding noise to the communication would allow exploring the strategies employed by animals to cope with adverse environments. For example, the Lombard effect and its neural underpinnings are still debated^30^. Also, a further important field of research in acoustic communication is the concept of turn-taking^35,36^, which could be dissected in detail using the described system.

## Technical details

### Sound acquisition

In each chamber, we mounted a microphone (Pro 42, Audio-Technica, Japan) with frequency response in the range 70–14 kHz and a sensitivity of 12.6 mV/Pa (− 38 dB re 1 V/Pa). The microphone signal was routed to a preamplifier (Q-Pre, SMPRO, Australia) with a 48 V phantom power supply. We set the gain close to maximum (40 dB), achieving an **input gain** ${g}_{i}$ (Fig. 3) of the microphone and preamplifier of ${g}_{i}$= 1.26 V/Pa.

**Figure 8:**
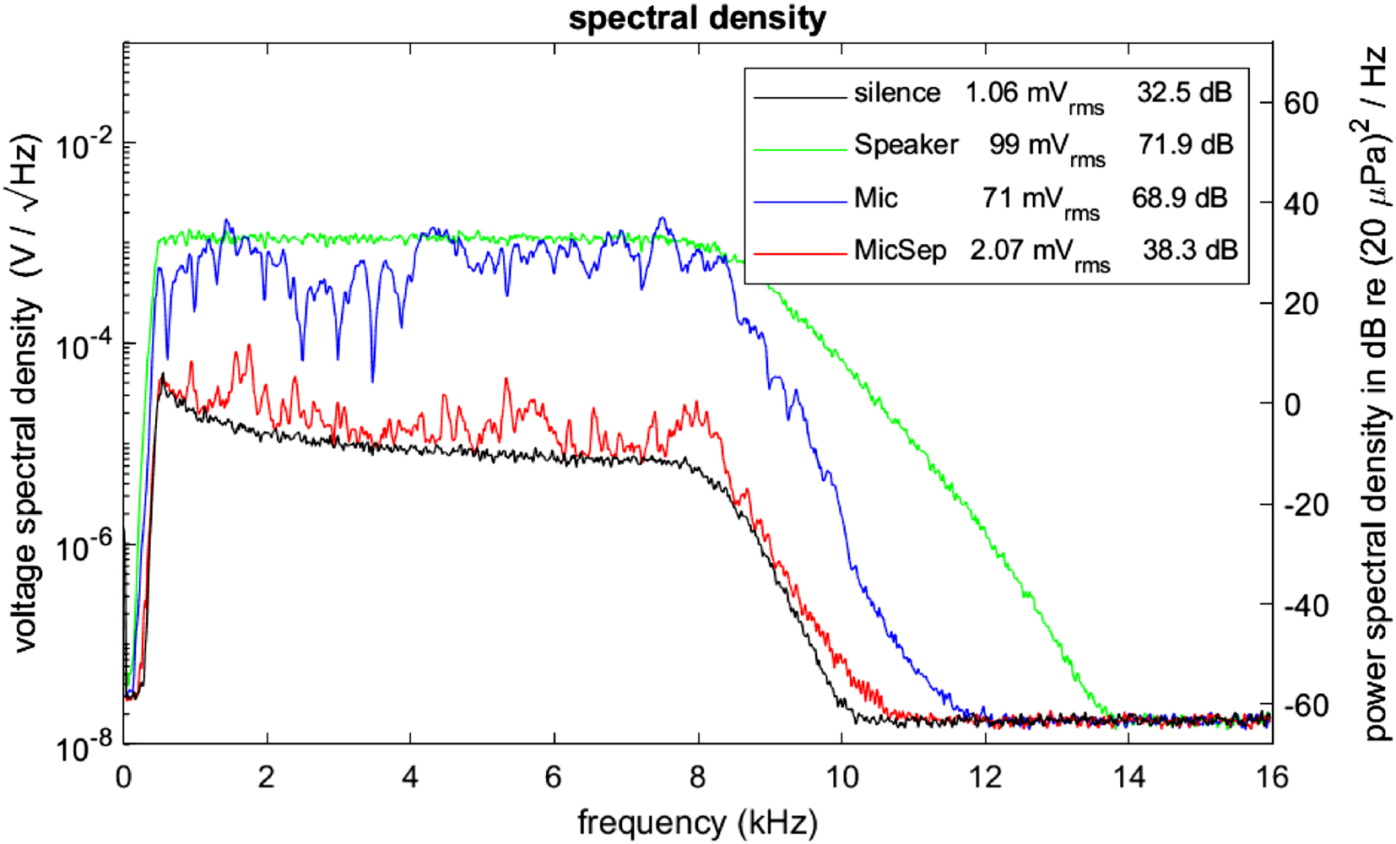
Power spectral densities of various input and output signals, demonstrating a roughly 30 dB echo attenuation. Green: white noise with a nominal 100 mV RMS amplitude played through the loudspeaker. Blue: raw microphone signal (Mic) with 3 dB lower amplitude. Red: the LMS filtered microphone signal (MicSep) with a roughly 30 dB reduced spectral density, close to the density of silence (black). The effect of the 500 Hz–8 kHz band-pass filter is visible by the cutoff. The legend indicates the overall RMS values and the corresponding sound pressure levels in dB re 20 µPa (unweighted).

The preamplifier outputs reaching in practice up to 0.65 V root mean square (RMS) amplitude were digitized in ± 10 V range with an analog input module (NI-9215, National Instruments, USA) at 96 kS/s and 16 bits/sample. On the FPGA, these signals were first decimated by a factor of 3 down to 32 kS/s using a moving average comb filter, serving also as an anti-aliasing filter. After that, signal samples were represented as fixed-point numbers with 20 bits precision and 4 bits integer range (± 8). The signals were digitally band-pass filtered with a passband from 500 to 8 kHz (Butterworth FIR filter of order 16 with stopband attenuation of 20 dB at 350 Hz and 10 kHz, and an equivalent noise bandwidth of 7.7 kHz). The resulting amplified, digitized, and conditioned **Mic signal** from an empty chamber had a total RMS noise of 1.06 mV, corresponding to 0.86 mPa or 32.5 dB (unweighted), Fig. 8.

### Sound reproduction

The digital (fixed point, 20-bit width, 4-bit integer, 32 kS/s) Speaker signal was band-pass filtered with the same bandpass filter we used for the Mic signal (500 Hz–8 kHz). This filter smoothed the sudden signal jumps created by either the opening of the squelch or the switching of the signal matrix. The Speaker signal was then up-sampled from 32 to 96 kS/s with an interpolating FIR filter and was converted at 16 bits precision to an analog signal in the range ± 10 V using an analog output module (NI-9263, National Instruments Corporation, USA). Thereafter, it was amplified by an audio amplifier (Alesis RA150, inMusicBrands, USA) and broadcast through a loudspeaker (HKS-6, Harman-Kardon, USA). For the bird’s safety and comfort, we limited the broadcast sound intensities to a maximum value of 85 dB at 25 cm above the bird’s head, which is within the natural range (Ritschard and Brumm, 2011).

### Chamber gain

The **chamber gain** from the Speaker signal to the Mic signal (Fig. 3) is defined as:

$${g}_{c}= {g}_{o}\cdot s\left({d}_{sm}\right)\cdot {g}_{i },$$

where ${g}_{o}$ is the **output gain** that relates the Speaker signal to the produced physical sound pressure, $s\left({d}_{sm}\right)$ is the transfer gain of acoustic waves in the air across the distance ${d}_{sm}$ between the loudspeaker and the microphone, and ${g}_{i}$ is the **input gain** of the Mic signal.

The chamber gain was strongly frequency dependent and could exceed 1 at certain frequencies (near 2, 4, and 8 kHz in Fig. 8, where the blue curve exceeds the green curve). To prevent feedback oscillations when two chambers are symmetrically connected, the product of their chamber gains, the closed-loop gain, must be below 1 for all frequencies. We found that this condition was fulfilled for ${g}_{c}=$ − 3 dB, because resonance frequencies were sufficiently different in the diverse chambers. Note that this limit of ${g}_{c}$ is highly conservative, purely a safety measure, because the echo attenuation described in the next section prevented feedback oscillations even when the product of chamber gains was higher than 1 (echo attenuated by 30 dB in each chamber).

Of interest is the transfer gain from one bird to another, which we express in terms of the virtual distance ${d}_{virt}$ between birds:

$$s\left({d}_{virt}\right)=s\left({d}_{bm}\right)\cdot {g}_{i}\cdot {g}_{o}\cdot s\left({d}_{sb}\right)={s\left({d}_{bm}\right)\cdot g}_{c}\cdot \frac{s\left({d}_{sb}\right)}{s\left({d}_{sm}\right)}.$$

Using the approximation of $s\left(d\right) \sim 1/d$ for spherical waves, we find

$${d}_{virt}\simeq \frac{1}{{g}_{c}} \frac{{d}_{bm} \cdot {d}_{sb}}{{d}_{sm}},$$

where ${d}_{bm}$ is the distance between bird and microphone, and ${d}_{sb}$ is the distance between the loudspeaker and the bird. The approximation in equation for ${d}_{virt}$ is evident in that the gain is frequency-dependent, the loudspeaker, the microphone, and the bird have non-isotropic directional characteristics, and waves do not propagate spherically because of reverberations. In an idealized setup in which the microphone, the loudspeaker, and the bird form an equilateral triangle (${d}_{bm}={d}_{sm}$) and all the chamber gains are set to ${g}_{c}=$ 0 dB, the receiving bird hears the sender bird as if the sender was positioned at the loudspeaker.

### Echo attenuation with an adaptive filter

Next, we describe the training procedure of the LMS filter. Let ${s}_{t}$ be the **Speaker signal** and $t=1, 2, 3$ the discrete-time index, i.e. the sample number. The **Speaker signal vector** ${{\varvec{s}}}_{t}$ of the last *L* Speaker*-*signal samples we write as

$${{\varvec{s}}}_{t}=\left[{s}_{t}, {s}_{t-1}, \dots , {s}_{t-L+1}\right].$$

We model the ***Mic signal*** ${m}_{t} (Fig. 3)$ as a linear function of the Speaker signal vector:

1 $${m}_{t}= {\varvec{h}} {{\varvec{s}}}_{t}+{e}_{t},$$

where the variable ${\varvec{h}}=[{h}_{1}, {h}_{2}, \dots , {h}_{L}]$ appearing in the scalar product represents the **LMS filter coefficients** and ${e}_{t}$ is the **error signal**. Minimizing the squared error signal ${E}_{t}=\frac{1}{2}{e}_{t}^{2}$ using an online gradient descent algorithm $\Delta {{\varvec{h}}}_{t}=-\mu {\nabla }_{{\varvec{h}}}{E}_{t}$ yields the iterative scheme:

2 $${{\varvec{h}}}_{t+1}= {{\varvec{h}}}_{t}+ \mu {{\varvec{s}}}_{t} {e}_{t},$$

where $\mu$ is the **adaptation rate**. In our graphical user interface, we let the user provide a normalized adaptation rate $M$ between 0 and 1, which sets the adaptation rate to.

$$\mu =M \frac{2}{L {\sigma }^{2}},$$

where $L$ is the filter length and ${\sigma }^{2}$ is the variance of the uniformly distributed white noise applied as Speaker signal ${s}_{t}$.

Four instances of this adaptive LMS filter were compiled on the FPGA (LabVIEW FPGA 18.0, Digital Filter Design Toolkit) with a filter of length $L$= 512 samples, which at 32 kS/s gives rise to an impulse response duration of 16 ms. We encoded the filter weights in the range ± 1 with 24-bits precision.

### Adaptation rate

**Figure 9:**
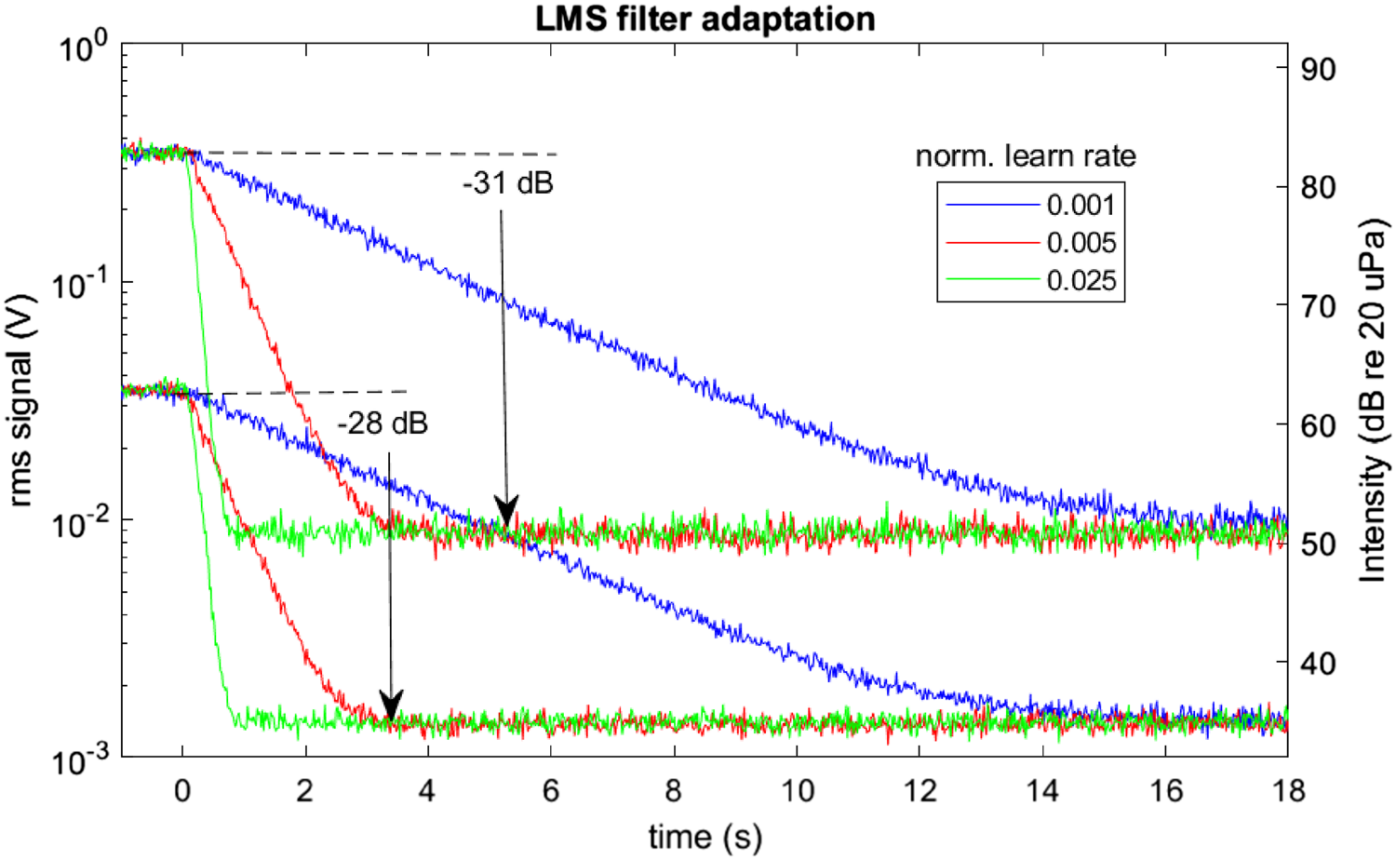
Rapid training of the LMS echo attenuation filter, shown for noise intensities of 63 dB (bottom three curves) and 83 dB (top three curves). In each case, three different normalized learning rates were tested: M = 0.001, 0.005, and 0.025. The filters achieved a suppression of roughly 30 dB. The limiting factor for the lower-intensity noise was the 32.5 dB noise floor of the microphone. The adaptation process works well with 65 dB noise, a learning time of 1–2 s, and a normalized learning rate of M = 0.025.

**Figure 10:**
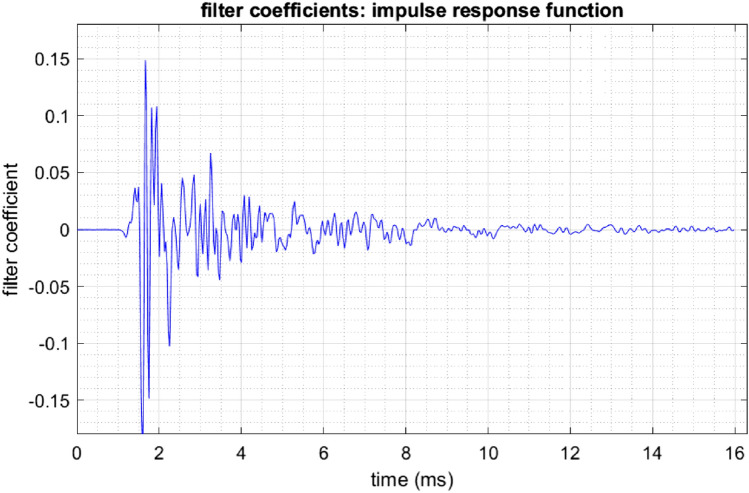
The impulse response (IR) function of the isolation chamber containing a plexiglass cage (between the loudspeaker and the microphone). The distance between the microphone and loudspeaker was about 50 cm, in agreement with the ~ 1.5 ms lag of the first IR extremum.

We measured the filter adaptation process for noise levels of 63 dB and 83 dB and normalized adaptation rates $M$ of 0.001, 0.005, and 0.025. We measured the performance of echo attenuation as the ratio of Mic and MicSep signal intensities measured during application of the white-noise stimulus. We achieved typical filter performance values of − 30 dB, with initial performance right after training as large as 40 dB. Based on the results shown in Figs. 8, 9 and 10, we chose for normal operation a noise level of 65 dB (45 mV), a normalized learning rate of 0.025, and an adaptation time of 1.5 s.

### Impulse response function

The filter coefficients constitute the impulse response (IR) function of the environment, Fig. 10. From this function, it is possible to estimate the distance between the loudspeaker and the microphone. Namely, the largest IR peak was located near 1.5 ms, corresponding to a distance of 49.5 cm (given an assumed speed of sound of 330 m/s). The subsequent peaks correspond to resonances produced by the plexiglass cage inside the chamber. These resonances decayed within about 8 ms, demonstrating that the filter duration of 16 ms was long enough to cancel out even the longest echoes.

The example in Fig. 10 shows that a plexiglass cage with parallel walls can introduce resonances that amplify or attenuate some frequencies by a difference of more than 20 dB, which imposes a detectable room characteristic on the vocal signature of birds. Birds kept in standard wire cages displayed fewer of these resonance peaks.

### Power estimation

Because the RMS computation is costly to implement on an FPGA, we computed mean square signals as estimates of signal power. On the FPGA, we implemented the mean square ${p}_{t}$ as a running average of the squared signal with an infinite impulse response filter (IIR):

$${p}_{t+1}={p}_{t}+\alpha \left({s}_{t}^{2}-{p}_{t}\right),$$

where $\alpha =1-exp\left(-\frac{\Delta t}{\tau }\right)$ sets the exponential decay and where $\Delta t=62.5 \mu \mathrm{s}$ is the sampling interval and $\tau$ the decay time constant, typically $\tau$ = 8 ms. We delayed the MicSep signal typically by the same time to avoid onset artifacts caused by the squelch.

### Animals and experiments

Zebra finches (*Taeniopygia guttata*) bred and raised in our colony (University of Zurich/ETHZ) were kept on a 14/10 h light/dark daily cycle, with food and water provided ad libitum. All experimental procedures were approved by the Cantonal Veterinary Office of the Canton of Zurich, Switzerland (license numbers ZH207/2013 and ZH077/17). All methods were carried out in accordance with relevant guidelines and regulations (Swiss Animal Welfare Act and Ordinance, TSchG, TSchV, TVV).

In all experiments, the LMS filter was trained each day right before the starting of the first recording session. Birds had also access to cuttlefish bone, sand bath, water bath, millet, and three perches. Before recording any data, we provided birds with 5 days habituation time in the setup, 1 h on the first day, and 1 additional hour each day until a maximum of 4 h was reached. After the habituation period, interaction channels were engaged during experiment sessions in the range from approximately 30 min to around 2.5 h, depending on the birds’ vocal activity. For the remainder of the day, the birds were housed in a large social cage.

For the experiment shown in Fig. 4, birds were each housed for 3 days (24 h/day) in a 24 × 24 × 20 cm^3^ plexiglass cage inside the isolation chambers. Birds could see each other through windows in the sidewalls of the chambers. For these experiments, the chambers were placed in an L-shape so that bird T in the middle chamber could see the two companion birds L and R and vice versa, but the companion birds could not see each other. The hierarchical communication network was enabled for 2–7 h a day, the rest of the time birds were acoustically isolated from one another.

In the experiment shown in Figs. 5, 6 and 7 with three males per replicate, the birds could move freely inside the recording chamber (60 × 60 × 60 cm^3^) that was equipped with a swing (although birds in Fig. 7b,c (blue curves) were housed in 39 × 23 × 39 cm^3^ plexiglass cages, as described in the previous paragraph). We noticed no severe limitation of our ICS under these more generous housing conditions, except that vocalizations tend to be both softer on one extreme and louder on the other (due to the more variable distance between bird and microphone), which implies a more restrictive squelching tradeoff (between suppressing soft vocalizations and residual echoes from loud vocalizations, Fig. 5). Following the 5-day habituation period, birds were placed into the setup for up to 4 h/day. We noticed that under these more transient housing conditions, vocalization rates tended to be smaller than in the 24 h/day setting (Fig. 4). To incentivize birds to vocalize in asymmetric cyclic networks, we played a female or male call roughly every 15–30 s to the top bird T. To minimize interference, in case of ongoing vocal interactions, playback was automatically delayed by 3.5 s.

### Cross-covariance analysis

We characterized vocal interactions between pairs of connected birds by the cross-covariance (CCV) function

$${CCV}_{A,B}(\tau )=\frac{1}{T}{\int }_{0}^{T}({\delta }_{A}(t)-\overline{{\delta }_{A}})({\delta }_{B}(t+\tau )-\overline{{\delta }_{B}})dt$$

of their mean-subtracted vocalization onset trains ${\delta }_{A},{\delta }_{B}$ and where $T$ denotes the duration of the session. We computed CCV functions up to a maximum lag of 2 s and smoothed them with a 300-ms Gaussian filter with standard deviation of 60 ms.

To assess the significance of CCV peaks, we shuffled the data using circular shifts during intervals of vocal activity. To identify these intervals, we first grouped call onsets of the responding bird into time intervals such that consecutive call onsets separated by less than 500 ms were grouped in the same interval. In case an interval was less than 2 s long, we extended it, to make the minimum interval duration 2 s. This grouping procedure was either running forward in time starting with the session beginning, or backward in time starting with the session end, with equal probability. On average, this grouping procedure resulted in $258\pm 350$ intervals per hour.

Within an interval, we circularly shifted the onsets by a common amount that we uniformly sampled in $[0,{t}_{i}]$, where ${t}_{i}$ is the interval duration. By repeating this random circular shifting procedure n = 200 times, we obtained a distribution of shuffled CCV functions. Significant CCV peaks had to exceed the standard deviation of this distribution by a factor of 3, corresponding to a p-value of roughly 0.01.

To compare CCV functions in a common plot, we normalized them as

$${CCV}_{\text{norm}}\left(\tau \right)=\frac{CCV\left(\tau \right)-{CI}_{\text{lower}}\left(\tau \right)}{{CI}_{\text{upper}}\left(\tau \right)-{CI}_{\text{lower}}\left(\tau \right)},$$

where the upper and lower confidence interval bounds ${CI}_{\text{upper}}$ and ${CI}_{\text{lower}}$ lied 3 standard deviations away from the mean of our random shuffle predictor.

## Data Availability

The code of the signal processing on the FPGA and the interactive user interface is provided as a LabVIEW project on GitLab (https://gitlab.ethz.ch/jrychen/birdconnect) and under the research data archive of ETH-Zürich under DOI (will follow).
